# Autophagy-Mediated Cellular Remodeling during Terminal Differentiation of Keratinocytes in the Epidermis and Skin Appendages

**DOI:** 10.3390/cells13201675

**Published:** 2024-10-10

**Authors:** Leopold Eckhart, Florian Gruber, Supawadee Sukseree

**Affiliations:** 1 Department of Dermatology, Medical University of Vienna, 1090 Vienna, Austria; 2 Christian Doppler Laboratory for Skin Multimodal Imaging of Aging and Senescence—SKINMAGINE, 1090 Vienna, Austria; 3 Center for Anatomy and Cell Biology, Medical University of Vienna, 1090 Vienna, Austria

**Keywords:** keratinocytes, cornification, autophagy, protease, hair, nail, sebaceous, keratin, apoptosis, skin barrier

## Abstract

The epidermis of the skin and skin appendages, such as nails, hair and sebaceous glands, depend on a balance of cell proliferation and terminal differentiation in order to fulfill their functions at the interface of the body and the environment. The differentiation of epithelial cells of the skin, commonly referred to as keratinocytes, involves major remodeling processes that generate metabolically inactive cell remnants serving as building blocks of the epidermal stratum corneum, nail plates and hair shafts. Only sebaceous gland differentiation results in cell disintegration and holocrine secretion. A series of studies performed in the past decade have revealed that the lysosome-dependent intracellular degradation mechanism of autophagy is active during keratinocyte differentiation, and the blockade of autophagy significantly alters the properties of the differentiation products. Here, we present a model for the autophagy-mediated degradation of organelles and cytosolic proteins as an important contributor to cellular remodeling in keratinocyte differentiation. The roles of autophagy are discussed in comparison to alternative intracellular degradation mechanisms and in the context of programmed cell death as an integral end point of epithelial differentiation.

## 1. Introduction

The epithelial compartment of the skin ensures the main functions of the skin, which are the protection of the body against exogenous insults and the prevention of water loss in a dry environment [1]. The epithelium and its specialized derivatives in skin appendages, such as hair, nails, sebaceous glands and sweat glands, are formed by keratinocytes. These cells establish stratified epithelia in which cell proliferation is generally limited to the innermost layer. Upon the exit from this layer, keratinocytes start so-called terminal differentiation. This process involves the expression of multiple genes that have functions unique for the outer epidermal layers or the mature parts of the hair and nails. Ultimately, keratinocytes are tightly packed with cytoskeletal proteins [2,3]. In the epidermis, keratinocytes secrete lipids and enzymes through the release of so-called lamellar bodies [4,5,6] in the granular layer. They also accumulate proteins that are cross-linked by transglutaminases to form a resilient protein scaffold underneath the cell membrane, which is known as the cornified envelope [7,8]. Another type of protein cross-linking, namely disulfide bond formation between cysteine residues of different proteins, is active in keratinocytes (trichocytes) forming the hair shaft and keratinocytes (onychocytes) forming the nail plate [9]. Ultimately, the cells undergo programmed cell death and become integrated into large structures consisting of interconnected dead cells, namely the cornified layer of the epidermis, hair shafts and nails [10,11,12]. A different path of keratinocytes differentiation is followed in sebaceous glands, which are connected to hair follicles, with the opening of the sebaceous duct into the upper hair canal. Sebaceous gland keratinocytes, known as sebocytes, accumulate lipids instead of structural proteins and conclude differentiation by cell disintegration to produce sebum. This type of differentiation underlies holocrine secretion [13,14].

The renewal of the stratum corneum, the growth of cornified hair and nails, and the production of sebum require the synthesis of proteins, lipids and other molecules [3,9,15]. The function of the cornified skin structures depends on specific keratins and other structural proteins, which are not present in proliferating keratinocytes or other cells [16,17]. Accordingly, they must be produced and correctly assembled within the time available for differentiation. The tightly regulated gene expression and post-translational modifications of proteins lead to the accumulation and packing of proteins in differentiating keratinocytes. Terminal differentiation is associated with an increase in the cellular keratin content to more than 80% of the total protein in corneocytes of the stratum corneum [18]. Likewise, hair and nail corneocytes are tightly packed with keratin filaments with an additional presence of keratin-associated proteins (KRTAPs) forming the matrix [9]. The cornified envelope of epidermal corneocytes comprises loricrin and other proteins encoded by genes clustered in the epidermal differentiation complex [19]. The terminal differentiation of keratinocytes involves also changes in the intercellular junctions, which initially allow cellular flexibility when individual keratinocytes move from the basal layer toward the skin surface or toward hair or nail [20]. Within the stratum corneum, desmosomes are stable until proteolysis separates corneocytes and facilitates desquamation. By contrast, hair and nail corneocytes remain tightly bound together. The regulation of intercellular connections, including a key role of tight junctions [21,22], has been characterized in recent years. Likewise, the intracellular remodeling during cornification has been investigated, and many critical subprocesses have been defined [11,23,24,25]. The present review focuses on the degradative processes, and particularly autophagy, that are active within differentiating keratinocytes.

Autophagy, literally meaning “self eating”, is the lysosome-dependent degradation of cellular components under the control of an evolutionarily conserved set of autophagy-related (Atg) genes [26]. Although chaperone-mediated autophagy and microautophagy are potential variants of autophagy [27,28], only the so-called macroautophagy is well documented in the skin and very likely is the main mechanism of autophagy relevant for keratinocytes [29,30,31]. Hence, all descriptions of “autophagy” refer to macroautophagy in this review. Autophagy begins with the formation of a phagophore or isolation membrane that encloses a part of the intracellular space to form a double-membraned vesicle, which is known as the autophagosome. This vesicle fuses with a lysosome and exposes its content to an array of proteases, lipases and other degradative enzymes present in the lysosome. The cargo of autophagosomes can comprise cytoplasmic molecules, entire organelles or fragments of organelles [32,33]. The degradation of the autophagosome cargo generates small molecules, such as amino acids being produced from proteins, which are released into the cytoplasm for incorporation into new macromolecules. In homeostasis, autophagy controls the recycling of substances, while its main function in stressed or infected cells is the removal of damaged cell components and exogenous material (xenophagy). When keratinocytes differentiate, the basal functions of autophagy are adapted to a dynamic, intrinsically controlled cell remodeling process.

We will first review the research evidence for the occurrence of autophagy in the various pathways of keratinocyte differentiation. Then, we will present a model for the function of autophagy as a mechanism for the degradation of intracellular material to make room for the products of synthetic processes. Finally, we will discuss the contribution of non-autophagic intracellular degradation mechanisms and the limitations of the current model.

## 2. Evidence for Autophagy during Keratinocyte Differentiation

### 2.1. Detection of Autophagosomes in Differentiated Keratinocytes

Autophagy is active in epithelial and other cells of the skin according to a variety of studies. Among several types of evidence for autophagy in tissues and cells [34], the observation of autophagosomes is considered reliable but technically difficult. Autophagic vesicles were demonstrated by transmission electron microscopy in the epidermis of newborn mice (postnatal day 3) [35], cultured keratinocytes [36], reconstructed human epidermis [37] and hair follicles [38,39]. Of note, the abundance of autophagosomes is low in cells with active autophagy because they rapidly fuse with lysosomes and subsequent undergo degradation. However, the attenuation of lysosomal function either by deletion of the lysosomal adaptor protein p18/LAMTOR1 or treatment with bafilomycin A1 increases the abundance of autophagosomes in keratinocytes [36].

Another approach for the detection of autophagy in situ is the utilization of green fluorescent protein (GFP)-tagged microtubule-associated protein 1 light chain 3 (MAP1LC3, short abbreviation LC3), which labels autophagosomes [40]. GFP-LC3 is incorporated into the autophagosome membrane through its LC3 part and allows detection by fluorescence microscopy based on its GFP domain. Using GFP-LC3 transgenic mice, labeled autophagosomes were detected in the epidermis [41,42], sebaceous glands [42,43,44], nail matrix [45], hair keratinocytes [46], sweat glands [47] and Merkel cells [48]. The abundance of autophagosomes appears to be higher in the upper layers of the epidermis than in the basal layer, which is paralleled by an increased detection of LC3 in the suprabasal layers [49].

### 2.2. Alteration of Keratinocyte Differentiation upon Suppression of Essential Autophagy-Related Genes

The comparison of autophagy-competent cells versus autophagy-deficient cells allows to determine (i) whether autophagy is active in normal cells so that its suppression leads to phenotypic differences and (ii) the specific roles of autophagy. The suppression of autophagy can be specifically achieved by the suppression of autophagy-related genes which have essential and exclusive functions in autophagy. Core factors of the molecular machinery of autophagy, such as Atg5, Atg7 and Atg16, are suitable targets in this regard [50]. The siRNA-mediated knockdown of autophagy-related gene expression has been used to reveal critical roles of Atg5-dependent autophagy in the organ culture of hair in vitro [39]. Similarly, knockdown of the WD repeat domain, phosphoinositide interacting 1 (WIPI1) and unc-51 like autophagy activating kinase 1 (ULK1) demonstrated autophagy in cultured keratinocytes [35].

To determine the roles of autophagy in complex organs such as the skin, gene deletions in the mouse model have been performed [50]. Mice lacking genes for the core machinery of autophagy either die in utero or perinatally so that an investigation of autophagy-deficient epidermis in comparison to autophagy-competent wild-type epidermis is not possible in this type of model [51,52]. However, skin was grafted from newborn *Atg7*-deficient mice onto immunodeficient mice and allowed to grow for one month [53]. *Atg7*-deficient skin grafts developed acanthosis and hyperkeratosis, and keratinocyte differentiation markers were reduced in comparison to skin grafted from autophagy-competent wild-type mice. Furthermore, hair growth was abnormal without autophagy [53]. The disadvantage of this model is that autophagy deficiency is not specific for epithelial cells and the phenotype may be partially attributable to other grafted cells.

To achieve the deletion of Atg genes (see below for details) specifically in epithelial skin cells, mice were generated in which Cre recombinase is expressed under the control of either the *Krt5* or *Krt14* promoter [54,55]. In these mice, Atg gene segments flanked by loxP sites are deleted in proliferating skin epithelial cells, abrogating autophagy also in cells that differentiate from these Krt5/Krt14-positive precursors. The roles of epithelial autophagy were determined by the deletion of *Atg5* [56,57,58], *beclin 1* (Becn1), which is the mammalian homolog of *Atg6* [59], *Atg7* [42,60,61,62], *Atg9a* [63], *Atg14* [59] and *Atg16l1* [31] in keratinocytes. Most of these studies detected deviations from the normal epithelial structure or function when autophagy was blocked. The deletion of *Atg7* and *Atg16l1* caused a thickening of the stratum corneum [31,41] which is likely a compensatory strategy to increase the barrier function of autophagy-deficient epidermis. All of the aforementioned studies came to the conclusion that the autophagy of epithelial skin cells is not essential for the viability of mice—at least under normal housing conditions. The survival of mice lacking epithelial autophagy is in line with the notion that the skin has a high tolerance to deviations from homeostasis [64], but it also indicates that the roles of autophagy should not be overestimated.

Aging and exposure to physical, chemical or other stressors have revealed functions of autophagy that are not apparent in the homeostatic young epidermis. For example, the secretory function of sweat glands was significantly decreased during the aging of autophagy-deficient mice [47]. Using the autophagy adaptor protein sequestosome (Sqstm1, also known as p62) as a marker, aberrations in protein turnover were prominently detected in long-lived epithelial cells, such as Merkel cells and the secretory cells of sweat glands [47,48] and less in rapidly differentiating cells [29]. Disabled macroautophagy is a hallmark of aging [65,66], and its role in the functional decline of skin epithelia requires further investigations [67]. Notably, autophagy contributes to the protection of keratinocytes against ultraviolet radiation and oxidative stress [31,57,61,68,69,70,71,72] and herpes simplex virus (HSV)-1 [73]. Furthermore, the initiation and growth of skin tumors was altered by the suppression of epithelial cell autophagy [31,61,69,74]. The pharmaceutical modulation of autophagic activity in the epidermis has been considered but has not been fully explored yet [29,75,76].

## 3. Autophagy Contributes to Intracellular Remodeling during Keratinocyte Differentiation

### 3.1. The Terminal Differentiation of Keratinocytes Leads to the Accumulation of Either Cytoskeletal Proteins or Lipids and the Removal of Many Other Cell Components

**Figure 1:**
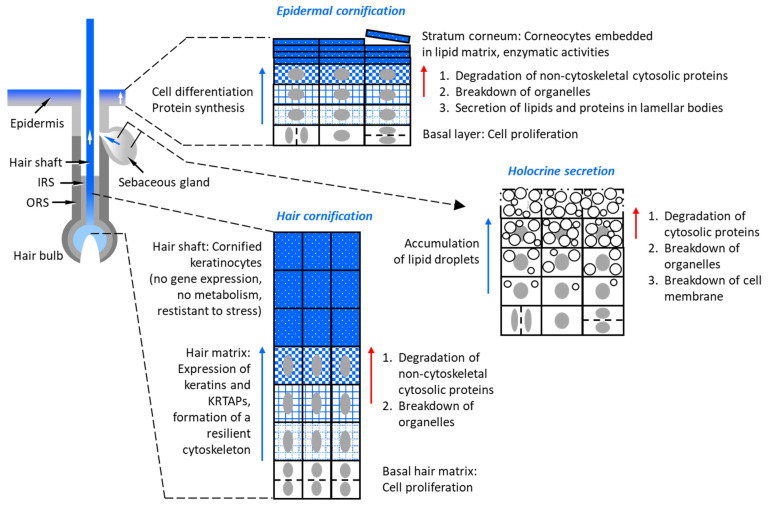
Degradation processes contribute to the terminal differentiation of keratinocytes in the epidermis, hair shafts and sebaceous glands. The increasing concentration of cytoskeletal proteins is highlighted by blue texture of cells (indicated by rectangles). The nucleus is depicted as a gray oval. In the schematic depiction of sebaceous gland cells, white circles indicate lipid droplets. IRS, inner root sheath; ORS, outer root sheath. This figure is adapted from a paper recently published by us under a Creative Commons CC-BY 4.0 license (open access) license [87].

The terminal differentiation of keratinocytes converts metabolically active cells that respond to internal and external stimuli into inert and stable corneocytes [3]. In contrast to living cells, which depend on organelles and a broad repertoire of receptors, enzymes, regulators etc., corneocytes require essentially only a massive and therefore stable cytoskeleton and a resilient envelope. In addition, large amounts of lipids are crucial for the functions of the stratum corneum and sebaceous glands. Lipids are secreted via lamellar bodies from keratinocytes of the granular layer to seal the intercellular space of the stratum corneum [4,5,77], whereas lipids accumulating in lipid droplets of sebocytes are released by holocrine secretion to become major components of sebum [13]. Importantly, the high concentrations of cytoskeletal proteins and lipids in terminally differentiated epithelial cells are not only achieved by the synthesis of these cell components, although this is certainly the main mechanism, but also by the removal of other cell components [3]. Indeed, there is histological, ultrastructural and biochemical evidence for the decrease or disappearance of various types of cellular material during keratinocyte differentiation [12,78] (Figure 1). Based on the notion of parallel synthesis and degradation of biomolecules, keratinocyte differentiation can be viewed primarily as a program for the remodeling of cells [3].

In this review, we discuss the contributions of autophagy to degradative processes in keratinocytes, mainly focusing on cell organelles and proteins. This simplified model does not integrate further differences between basal and differentiated epithelial skin cells, which are discussed elsewhere [21,79,80,81,82]. To present the basic concept, we describe evidence and hypotheses pertaining to epithelial cell differentiation in homeostatic or non-stressed skin. Any aberrant decrease or increase in autophagy in skin diseases has been reported and reviewed previously [83,84,85,86].

### 3.2. Autophagic Degradation of Cell Organelles during Keratinocyte Differentiation

Autophagy is able to degrade a broad variety of organelles. Specific receptors for proteins present on the surface of different organelles are utilized for the enclosure of organelles, or parts thereof, by phagophores [88] to initiate autophagy. The terminal differentiation of keratinocytes involves the rearrangement, destruction and removal of many if not all organelles [89]. Corneocytes of normal epidermis do not contain detectable organelles, whereas corneocytes of lesional skin in psoriasis and several other diseases contain histologically visible nuclear remnants, which is a condition known as parakeratosis [90]. In hard cornification within the hair shaft, nuclear remnants and remnants of other organelles are regularly found at the ultrastructural level [9,91], but these remnants are partially degraded [92].

**Figure 2:**
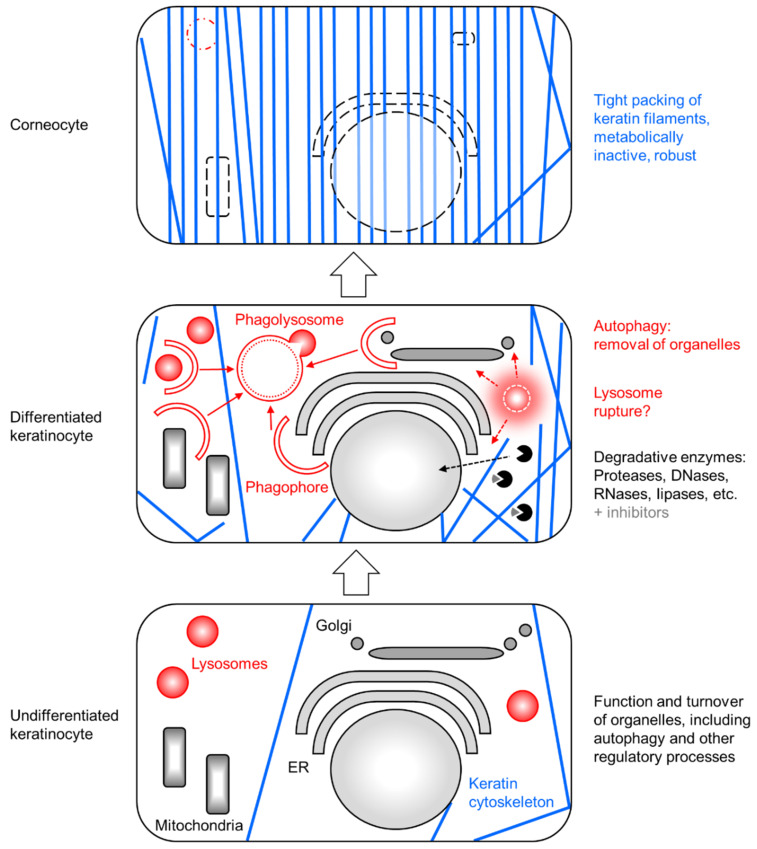
Cell remodeling by autophagy in keratinocytes. Three cells representing differentiation stages of keratinocytes are schematically depicted. The degradation of organelles in differentiated keratinocytes involves an interplay between autophagy and degradative enzymes that are either released from lysosomes upon rupture of these organelles or localize to other organelles or the cytosol, where some of them are bound by proteinaceous inhibitors. ER, endoplasmic reticulum.

Upon the molecular characterization of the molecular machinery of autophagy in mammalian cells [32,34,50], research tools and genetically defined mouse models could be generated to test the hypothesis that autophagy degrades organelles in differentiated keratinocytes. Some studies explored autophagy in the removal of particular organelles, whereas other studies abolished autophagy in general in keratinocytes. The working model of these studies is depicted in Figure 2. Alternative mechanisms for the degradation of cell content, such as the action of degradative enzymes produced in the cytosol or released by lysosome rupture, are included in this figure and will be discussed in Section 4.

#### 3.2.1. Autophagic Degradation of the Nucleus (Nucleophagy) in Keratinocytes

Several studies have suggested that autophagy is involved in the breakdown of the nucleus during terminal differentiation [35,42,93]. Autolysosomes containing nuclear proteins were detected in keratinocytes differentiating in vitro, and LC3-positive vesicles were located close to the nucleus in granular layer keratinocytes [35]. Knockdown of the autophagy genes *WIPI1* and *ULK1* decreased nucleophagy-like features, and a decrease in LC3 correlated with the aberrant retention of the nucleus in the stratum corneum of psoriatic lesions [35]. Within the degradation process of the nucleus, the protein kinase AKT1 triggers the dispersal of the nuclear lamina and shrinkage of the nucleus [94].

Recently, the breakdown of the nucleus was linked to vesicle-associated membrane protein 2 (VAMP2), which is associated with the autophagy proteins LC3 and WIPI1 in differentiated keratinocytes [93]. VAMP2 also associates with the focal adhesion kinase family interacting protein of 200 kDa (FIP200), also known as RB1-inducible coiled-coil protein 1 (RB1CC1), which contributes to the targeting of autophagosomes as a component of the ULK1 complex [93]. Mice lacking FIP200 in the epidermis developed epidermal hyperplasia and parakeratosis and lost hair [93]. As the deletion of VAMP2 causes perinatal lethality, the late embryonic skin of knockout mice and the cells grafted onto immunodeficient mice were investigated. Both in the embryonic development and in skin grafts, the degradation of the nucleus was decreased but not entirely blocked [93]. VAMP2 and FIP200 are not exclusively active in the control of autophagy. Therefore, further studies are necessary to determine their role in epidermal enucleation.

#### 3.2.2. Autophagic Degradation of Mitochondria (Mitophagy) in Keratinocytes

Mitochondria are degraded when keratinocytes of the granular layer undergo cornification. The initial presence of mitochondria and their subsequent disappearance can be visualized by mitochondria-specific dyes [89] and the immunolabeling of mitochondrial proteins [95]. Furthermore, mitochondrial fragments were detected within lysosomes in the epidermal granular layer of rat skin [78]. An elegant investigation of the spatiotemporal relationships of mitochondria, autophagosomes and lysosomes in human epidermal equivalent models showed a fragmentation of mitochondria, partial co-localization with autophagosomes and depolarization of mitochondria that escaped degradation by autophagy [89].

Autophagy can degrade mitochondria or fragments thereof in a non-selective manner as part of so-called bulk autophagy or selectively via autophagy receptors that bind mitochondria. Several mitochondria-targeting selective autophagy receptors, such as BCL2 and adenovirus E1B 19-kDa-interacting protein 3 (BNIP3), BNIP3-like (BNIP3L), BCL2L13 and autophagy and beclin 1 regulator 1 (AMBRA1), have been identified [8]. BNIP3 was reported to be important both for the maintenance and the differentiation of epidermal keratinocytes [95]. Subsequently, BNIP3L, also known as NIX, was demonstrated to initiate mitochondrial fragmentation [82]. The depletion of BNIP3L suppressed the removal of mitochondria [82]. This process is partly regulated by a micro-RNA and linked to UVB-induced senescence with implications on changes of epidermal differentiation during aging [96]. Very recently, a microautophagy-like process was shown to exist for the removal of the mitochondrial inner membrane [97], but its potential role in keratinocytes remains to be investigated.

In hair keratinocytes (trichocytes), mitochondria are deactivated by the loss of their membrane potential already in an early phase of differentiation [9,12]. The loss of mitochondrial membrane potential coincides with the loss of vesicles that can be stained with lysotracker red [98]. Studies in wool follicles have shown that mitochondria are partly degraded by autophagy and partly remain detectable as fragments in the mature hair shaft [91]. In mice lacking Atg7-dependent autophagy in keratinocytes, mitochondrial proteins are elevated 2.7-fold in hair shafts, suggesting that autophagy is required to remove most of the mitochondrial mass during normal hair cornification [87].

#### 3.2.3. Autophagic Degradation of Other Organelles in Keratinocytes

In addition to the nucleus and mitochondria, other organelles such as the endoplasmic reticulum (ER), the Golgi apparatus and lysosomes disappear during the cornification of keratinocytes. These organelles have been identified as targets of selective autophagy in other cell types [88,99]. Ultrastructural investigations of granular layer keratinocytes have suggested that the ER undergoes fragmentation before the nucleus is degraded [42]. To the best of our knowledge, the paths of degradation of the ER and the Golgi have not been ultrastructurally characterized in keratinocytes. However, it is important to note that the trans-Golgi network is linked to the lamellar bodies of the granular layer in the epidermis. The application of three-dimensional electron microscopy methodologies revealed that the Golgi apparatus and lamellar bodies form a tubuloreticular membrane network that is continuous with the plasma membrane of stratum granulosum cells [5]. Accordingly, a part of the material of the Golgi apparatus may be secreted into the intercellular space during epidermal cornification rather than being degraded intracellularly.

The cornification of hard skin appendages is not associated with lamellar bodies, indicating that the breakdown by autophagy may be a pathway of degradation of the Golgi. This hypothesis predicts that Golgi proteins and autophagy receptors of the Golgi should accumulate in the absence of autophagy. Indeed, the ATG8 homolog GABA type A receptor associated protein like 2 (GABARAPL2), which locates in the membranes of the Golgi and autophagosomes, was 6-fold elevated in hair shafts of epithelial *Atg7*-deficient mice [87]. Similarly, the calcium-binding and coiled-coil domain-containing protein (CALCOCO1), which mediates selective autophagy of the Golgi [100], was elevated in the cornified nails of epithelial *Atg7*-deficient mice [45]. Similarly, three receptors for selective autophagy of the endoplasmic reticulum, namely the protein encoded by testis-expressed gene 264 (TEX264) [101], reticulon-3 (RTN3) [102], and atlastin-3 (ATL3) [103], were elevated in the nails when Atg7-dependent autophagy was blocked [45].

Lysosomes are essential for the execution of autophagy, but they can also be targets of autophagy (lysophagy) [104]. Recently, spartin (SPART), also known as SPG20, was identified as a lysosomal damage sensor that initiates the ubiquitination of lysosomal surface proteins and the subsequent autophagic degradation of lysosomes [105]. At present, the extent of lysophagy in differentiated keratinocytes is not known, but a role of lysophagy in sebocytes was suggested by results of studies using *Atg7* and *Dnase2a* knockout mice [14], as will be discussed in Section 4.3.

### 3.3. Autophagic Degradation of Cytoplasmic Proteins in Keratinocytes

Comprehensive analyses of the autophagy-dependent degradome were performed for nails [45] and hair shafts [87]. Both studies revealed that the suppression of autophagy leads to an increase in a wide variety of cytosolic proteins. The mRNAs corresponding to some of the presumable autophagy substrate proteins were not elevated [45], indicating that the increased protein abundance was not caused by increased gene expression but rather by a decrease in degradation in the absence of autophagy. The accumulation of structurally and functionally diverse enzymes and other proteins suggested that these proteins were degraded by bulk autophagy in normal keratinocytes.

Interestingly, protein complexes, such as the CCT/TriC chaperonin and proteasomes, displayed particularly strong increases in abundance when autophagy was blocked [45,87]. The abundance of chaperonin increased 9.8-fold in nails and 12.6-fold in hair shafts. Proteasomes were elevated 9.6-fold in nails and 9.7-fold in hair shafts. The parallel accumulation of many or all subunits of these multi-protein complexes essentially excluded random fluctuations as the cause of the observed elevation [45,87] and, more importantly, suggested a specific dependence of these complexes on degradation by autophagy [106,107]. A possible explanation is that upon ubiquitination, non-complexed proteins can be targeted by both autophagy and proteasomes, whereas large complexes depend on autophagy for degradation. Interestingly, the relative accumulation of another multi-component complex, the ribosome [108], was more pronounced in hair shafts (3.9-fold) than in nails (1.9-fold) of mice lacking *Atg7* in skin epithelial cells [45,87].

**Figure 3:**
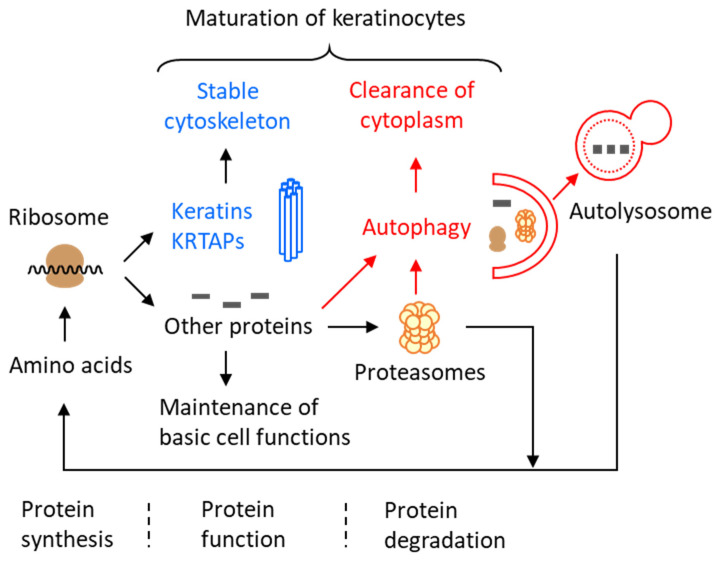
Protein degradation by autophagy in differentiating keratinocytes. This schematic is based on results of studies of nail plates and hair shafts from wild-type and epithelial *Atg7*-deficient mice. KRTAPs, keratin-associated proteins. This figure is adapted from a paper recently published by us under a Creative Commons CC-BY 4.0 license (open access) license [87].

The elevation of non-cytoskeletal proteins upon the deletion of *Atg7* was compensated by a decreased relative abundance of keratins and keratin-associated proteins in cornified nails and hair shafts. At the mRNA level, the expression of keratins was not altered in the nail matrix [45]. The decrease in the cytoskeleton content may be caused by reduced efficiency of the post-transcriptional synthesis steps, which is possibly due to the reduced supply of amino acids in the absence of an autophagy-dependent breakdown of non-cytoskeletal proteins. In an alternative scenario, the synthesis rate of keratins is not altered, but the increased retention of non-cytoskeletal proteins leads to a relative decrease in the keratins’ share of the total proteome (Figure 3).

## 4. Non-Autophagic Degradation Mechanisms Are Active besides Autophagy in Differentiated Keratinocytes

### 4.1. Proteasomal Degradation of Proteins in Keratinocytes

Despite several lines of evidence for roles of autophagy in intracellular degradation processes during keratinocyte differentiation, alternative mechanisms of degradation are also active and may be even more important than autophagy for specific aspects of cornification or holocrine secretion. The ubiquitin–proteasome system is a ubiquitous degradation route for intracellular proteins [109]. Proteasomes are active in basal and supra-basal keratinocytes, and several lines of evidence suggest that they contribute to cellular remodeling. The ubiquitin ligase kelch like family member 24 (KLHL24) is expressed in keratinocytes and other cells [110]. In the epidermis and the hair follicle, KLHL24 targets keratins K14 and K15, respectively, for degradation by the proteasome. Mutations of KLHL24 lead to the aberrant stabilization of the enzyme. Subsequently, excessive degradation of their target keratins causes epidermolysis bullosa and alopecia [111,112]. Similarly, KLHL16 targets keratins K6, K16, and K17 for proteasomal degradation. These keratins are predominantly expressed in stressed or migrating keratinocytes, which remodel their keratin cytoskeleton [113].

Proteasome maturation protein (POMP) is a chaperone for the maturation of proteasomes. A mutation of the POMP gene causes an aberrant distribution of proteasome subunit α7 in the upper epidermis, proteasome insufficiency in differentiated keratinocytes, and a disease called keratosis linearis with ichthyosis congenita and sclerosing keratoderma (KLICK) syndrome [114]. Proteasomal degradation has been reported to target the important skin barrier protein filaggrin [115]. However, the impairment of proteasomes induces an unfolded protein response, which may alter the transcriptional program of keratinocyte differentiation including the expression of filaggrin [116]. Thus, further studies are necessary to determine the extent of proteasomal degradation in differentiated keratinocytes.

### 4.2. Enzymatic Degradation of Proteins and DNA in Differentiated Keratinocytes

Several enzymes are able to degrade intracellular molecules during normal metabolism and as part of cellular quality control processes. When cells are induced to undergo apoptosis, which is a ubiquitous mechanism of programmed cell death, an efficient intracellular degradation program involving proteases and nucleases is initiated. Classical pro-apoptotic proteases of the caspase family are not active in differentiating keratinocytes [117]. Instead, the keratinocyte-associated caspase-14, which is phylogenetically related to pro-inflammatory caspases, is fully activated in differentiated keratinocytes of the epidermis, where it contributes to the proteolytic processing of filaggrin [118,119]. A plethora of proteases, many of which are controlled by specific inhibitors, is present in differentiated keratinocytes [120,121]. These proteases contribute to the degradation of cellular material during cornification and may remain active, to some extent, in corneocytes, besides the well-established extracellular proteolytic activity leading to the desquamation of corneocytes.

Among other types of catabolic enzymes, we mention here only DNA-degrading enzymes (DNases) because (i) they play specific roles in keratinocyte differentiation and (ii) their action is modified by autophagy. Terminal differentiation of keratinocytes in the epidermis, hair follicles and the nail apparatus is associated with the transcriptional upregulation of DNase1L2, which is a skin-specific DNase [92]. DNase1L2 degrades nuclear and mitochondrial DNA in hair and nail keratinocytes [92] and contributes to epidermal DNA degradation [122]. Although DNase1L2 is catalytically active in an acidic milieu, it is not active in lysosomes. Therefore, DNase1L2-dependent DNA breakdown is not part of autophagy. Proteomic analyses have rather shown that DNase1L2 is elevated in the hair shafts [87] and nails of epithelial autophagy-deficient mice without being transcriptionally upregulated [45], suggesting that it is a substrate of autophagy in normal hard cornification. Accordingly, DNA degradation by DNase1L2 either occurs before the enzyme is degraded or it depends on a fraction of DNase1L2 that is spared from autophagy. DNase1L2 cooperates with the exonuclease TREX2 [123] and DNase 2 [122], which is a lysosomal enzyme that will be discussed in the next section.

### 4.3. Degradative Enzymes Released from Lysosomes

Lysosomes play critical roles in keratinocytes differentiation [85,124,125,126]. However, not all roles of lysosomes depend on autophagy [127]. Lysosomes are not static but rather subjected to new synthesis and degradation (Section 3.2.3). Lipid peroxidation as well as changes in lipid composition, crystals and bacteria can cause the permeabilization or rupture of the lysosomal membrane, leading to the release of lysosomal enzymes into the cytosol. This defect can lead to cell death and tissue inflammation [127,128]. The live imaging of granular layer keratinocytes revealed that acidic vesicles, equivalent to lysosomes, displayed a decline of mobility and a subsequent loss of the LysoTracker Red (Thermo Fisher Scientific, Waltham, MA, USA) signal, suggesting an increase in lysosomal membrane permeability when keratinocytes underwent cornification [89].

DNase 2, the lysosomal DNase with an acidic pH optimum, cooperates with DNase1L2 in the degradation of nuclear DNA in differentiated keratinocytes of the mouse epidermis. The co-deletion of *Dnase2a* and *Dnase1l2* prevented the breakdown of nuclear DNA and led to parakeratosis [122]. It is conceivable that DNase 2 is part of an autophagic degradation route of nuclear DNA that is complemented by extra-lysosomal DNA degradation through DNase1L2. However, it is also possible that DNase 2 is released from lysosomes to degrade DNA upon acidification of the cytosol in terminally differentiated keratinocytes. Notably, DNase 2 remains intact and catalytically active after cornification and degrades even exogenous DNA on the skin surface [129].

The investigation of sebaceous glands has provided results that clearly argue for a non-autophagic role of the lysosomal enzyme DNase 2 [14]. In sebaceous glands, the suppression of autophagy causes two histologically visible alterations. First, the differentiated sebocytes in the middle of the gland become aberrantly eosinophilic when either *Atg5* or *Atg7* are deleted in epithelial cells [14,56]. The increased affinity for eosin indicates an altered composition of sebocytes, although the changes responsible for binding eosin have not been determined so far. The second phenotype of autophagy-deficient sebaceous glands is the disappearance of the nucleus in cells close to the basal layer [14,56], suggesting that the degradation of the nucleus is initiated and completed in an aberrantly early stage of sebocyte differentiation. This consequence of autophagy deficiency does not exclude that autophagy contributes to nuclear degradation, but it definitely refutes the hypothesis that autophagy is necessary for the breakdown of the nucleus in sebocytes. The premature degradation of nuclear DNA was suppressed when *Dnase2a* was deleted together with *Atg7* [14], demonstrating that the lysosomal DNase was required for the autophagy-independent degradation of nuclear DNA. The deletion of *Dnase2a* also suppressed nuclear DNA breakdown in autophagy-competent sebaceous glands [14].

We have presented the following hypothesis to explain the interactions of autophagy and DNase 2 during sebocyte differentiation [14]). Autophagy affects lysosomes by delivering cellular material into these vesicles and by degrading defective lysosomes (lysophagy) [128]. In sebaceous glands that lack autophagy, lysosomes are more likely to become dysfunctional and less likely to be removed. These effects increase the rate of lysosomal rupture, which leads to the release of proteases, nucleases, and other degradative enzymes into the cytosol [127,130]. Assuming that the cytosolic pH of sebocytes drops before holocrine secretion in a similar way as in epidermal keratinocytes before cornification [10,131], DNase 2 is able to hydrolyze DNA outside of lysosomes, including nuclear DNA in terminally differentiated sebocytes.

## 5. Conclusions: Degradation and Synthesis of Proteins Are Complementary Processes Required for Cellular Remodeling during Terminal Differentiation of Keratinocytes

**Figure 4:**
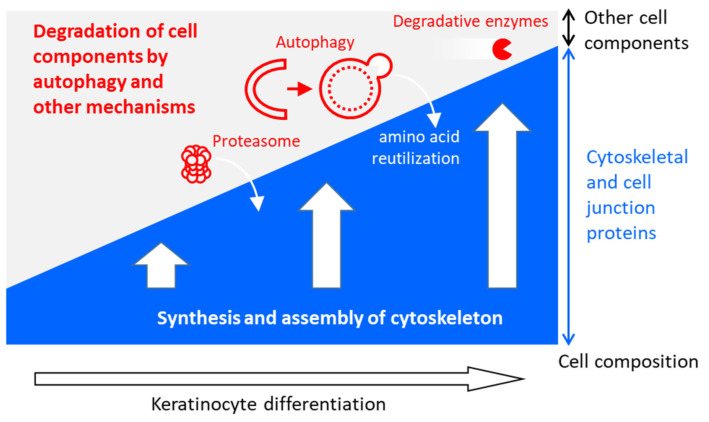
The synthesis of the cytoskeleton is accompanied by the degradation of other cell components during the terminal differentiation of keratinocytes.

In conclusion, we propose a model for autophagy as a contributor to the terminal differentiation of keratinocytes, as depicted in Figure 4. In this model, autophagy and other mechanisms are used by keratinocytes to degrade cell components that do not support or that even disturb the function of the end products of differentiation, namely corneocytes in the epidermis, nail or hair. The breakdown of proteins in lysosomes but also in the cytosol produces amino acids that can be used for the synthesis of cytoskeletal proteins. Moreover, the degradation of cytoplasmic proteins and organelles gives room for the extension of the cytoskeleton, which is necessary for the maturation of corneocytes. Some of these degradative processes are likely also active in differentiating sebocytes, although the cytoskeleton is less important and the formation of lipid droplets is more important there.

This model contains several uncertainties and leaves open questions, such as the following. By which mechanisms are proteins targeted to degradation or cross-linking during cornification? How is the intracellular remodeling in the upper granular layer linked to the increase in the cytosolic calcium concentration, which is required for the activity of transglutaminases [7,132]? What are the mechanistic commonalities and differences between terminal differentiation of keratinocytes in the epidermis, hard skin appendages and sebaceous glands? To what extent do deviations in autophagy and other cellular degradation processes contribute to skin pathologies [86,125,133,134,135,136,137,138]? Further targeted gene knockout experiments, in vivo imaging studies of molecules and organelles, and other studies are necessary to provide more insights into the roles of autophagy in keratinocyte differentiation.

## References

[1] Hsu Y.C., Fuchs E. (2022). Building and maintaining the skin. Cold Spring Harb. Perspect. Biol..

[2] Watt F.M. (1989). Terminal differentiation of epidermal keratinocytes. Curr. Opin. Cell Biol..

[3] Fuchs E. (1990). Epidermal differentiation: The bare essentials. J. Cell Biol..

[4] Wertz P. (2018). Epidermal lamellar granules. Skin Pharmacol. Physiol..

[5] den Hollander L., Han H., de Winter M., Svensson L., Masich S., Daneholt B., Norlén L. (2016). Skin lamellar bodies are not discrete vesicles but part of a tubuloreticular network. Acta Derm. Venereol..

[6] Raymond A.A., Gonzalez de Peredo A., Stella A., Ishida-Yamamoto A., Bouyssie D., Serre G., Monsarrat B., Simon M. (2008). Lamellar bodies of human epidermis: Proteomics characterization by high throughput mass spectrometry and possible involvement of CLIP-170 in their trafficking/secretion. Mol. Cell. Proteom..

[7] Candi E., Schmidt R., Melino G. (2005). The cornified envelope: A model of cell death in the skin. Nat. Rev. Mol. Cell Biol..

[8] Matsui T., Amagai M. (2015). Dissecting the formation, structure and barrier function of the stratum corneum. Int. Immunol..

[9] Harland D.P., Plowman J.E. (2018). Development of hair fibres. Adv. Exp. Med. Biol..

[10] Matsui T., Kadono-Maekubo N., Suzuki Y., Furuichi Y., Shiraga K., Sasaki H., Ishida A., Takahashi S., Okada T., Toyooka K. (2021). A unique mode of keratinocyte death requires intracellular acidification. Proc. Natl. Acad. Sci. USA.

[11] Matsui T. (2023). Epidermal barrier development via corneoptosis: A unique form of cell death in stratum granulosum cells. J. Dev. Biol..

[12] Jones L.A., Harland D.P., Jarrold B.B., Connolly J.E., Davis M.G. (2018). The walking dead: Sequential nuclear and organelle destruction during hair development. Br. J. Dermatol..

[13] Schneider M.R., Paus R. (2010). Sebocytes, multifaceted epithelial cells: Lipid production and holocrine secretion. Int. J. Biochem. Cell Biol..

[14] Fischer H., Fumicz J., Rossiter H., Napirei M., Buchberger M., Tschachler E., Eckhart L. (2017). Holocrine secretion of sebum is a unique DNase2-dependent mode of programmed cell death. J. Investig. Dermatol..

[15] Perrin C., Langbein L., Schweizer J. (2004). Expression of hair keratins in the adult nail unit: An immunohistochemical analysis of the onychogenesis in the proximal nail fold, matrix and nail bed. Br. J. Dermatol..

[16] McLean W.H., Moore C.B. (2011). Keratin disorders: From gene to therapy. Hum. Mol. Genet..

[17] Ortner-Tobider D., Trafoier T., Moosbrugger-Martinz V., Tollinger S., Gruber R., Schmuth M. (2024). Keratin variants in monilethrix. Br. J. Dermatol..

[18] Sun T.T., Green H. (1978). Keratin filaments of cultured human epidermal cells. Formation of intermolecular disulfide bonds during terminal differentiation. J. Biol. Chem..

[19] Henry J., Toulza E., Hsu C.Y., Pellerin L., Balica S., Mazereeuw-Hautier J., Paul C., Serre G., Jonca N., Simon M. (2012). Update on the epidermal differentiation complex. Front. Biosci. (Landmark Ed.).

[20] Peskoller M., Bhosale A., Göbel K., Löhr J., Miceli S., Perot S., Persa O., Rübsam M., Shah J., Zhang H. (2022). How to build and regenerate a functional skin barrier: The adhesive and cell shaping travels of a keratinocyte. J. Investig. Dermatol..

[21] Yokouchi M., Kubo A. (2018). Maintenance of tight junction barrier integrity in cell turnover and skin diseases. Exp. Dermatol..

[22] Atsugi T., Yokouchi M., Hirano T., Hirabayashi A., Nagai T., Ohyama M., Abe T., Kaneko M., Zouboulis C.C., Amagai M. (2020). Holocrine secretion occurs outside the tight junction barrier in multicellular glands: Lessons from claudin-1-deficient mice. J. Investig. Dermatol..

[23] Norlén L., Lundborg M., Wennberg C., Narangifard A., Daneholt B. (2022). The skin’s barrier: A cryo-EM based overview of its architecture and stepwise Formation. J. Investig. Dermatol..

[24] Méchin M.C., Simon M. (2023). Deimination in epidermal barrier and hair formation. Philos. Trans. R. Soc. Lond. B Biol. Sci..

[25] Huang L.Y., Li S.T., Lin S.C., Kao C.H., Hong C.H., Lee C.H., Yang L.T. (2023). Gasdermin A is required for epidermal cornification during skin barrier regeneration and in an atopic dermatitis-like model. J. Investig. Dermatol..

[26] Yamamoto H., Zhang S., Mizushima N. (2023). Autophagy genes in biology and disease. Nat. Rev. Genet..

[27] Kaushik S., Cuervo A.M. (2018). The coming of age of chaperone-mediated autophagy. Nat. Rev. Mol. Cell Biol..

[28] Kuchitsu Y., Taguchi T. (2024). Lysosomal microautophagy: An emerging dimension in mammalian autophagy. Trends Cell Biol..

[29] Eckhart L., Tschachler E., Gruber F. (2019). Autophagic control of skin aging. Front. Cell Dev. Biol..

[30] Liu C., Gu L., Ding J., Meng Q., Li N., Dai G., Li Q., Wu X. (2021). Autophagy in skin barrier and immune-related skin diseases. J. Dermatol..

[31] Van Hove L., Toniolo A., Ghiasloo M., Lecomte K., Boone F., Ciers M., Raaijmakers K., Vandamme N., Roels J., Maschalidi S. (2023). Autophagy critically controls skin inflammation and apoptosis-induced stem cell activation. Autophagy.

[32] Mizushima N., Komatsu M. (2011). Autophagy: Renovation of cells and tissues. Cell.

[33] Morishita H., Mizushima N. (2019). Diverse cellular roles of autophagy. Annu. Rev. Cell Dev. Biol..

[34] Klionsky D.J., Petroni G., Amaravadi R.K., Baehrecke E.H., Ballabio A., Boya P., Bravo-San Pedro J.M., Cadwell K., Cecconi F., Choi A.M.K. (2021). Autophagy in major human diseases. EMBO J..

[35] Akinduro O., Sully K., Patel A., Robinson D.J., Chikh A., McPhail G., Braun K.M., Philpott M.P., Harwood C.A., Byrne C. (2016). Constitutive autophagy and nucleophagy during epidermal differentiation. J. Investig. Dermatol..

[36] Soma-Nagae T., Nada S., Kitagawa M., Takahashi Y., Mori S., Oneyama C., Okada M. (2013). The lysosomal signaling anchor p18/LAMTOR1 controls epidermal development by regulating lysosome-mediated catabolic processes. J. Cell Sci..

[37] Cau L., Takahara H., Thompson P.R., Serre G., Méchin M.C., Simon M. (2019). Peptidylarginine deiminase inhibitor Cl-amidine attenuates cornification and interferes with the regulation of autophagy in reconstructed human epidermis. J. Investig. Dermatol..

[38] Orwin D.F. (1976). Acid phosphatase distribution in the wool follicle. I. Cortex and fiber cuticle. J. Ultrastruct. Res..

[39] Parodi C., Hardman J.A., Allavena G., Marotta R., Catelani T., Bertolini M., Paus R., Grimaldi B. (2018). Autophagy is essential for maintaining the growth of a human (mini-)organ: Evidence from scalp hair follicle organ culture. PLoS Biol..

[40] Mizushima N., Yamamoto A., Matsui M., Yoshimori T., Ohsumi Y. (2004). In vivo analysis of autophagy in response to nutrient starvation using transgenic mice expressing a fluorescent autophagosome marker. Mol. Biol. Cell..

[41] Rossiter H., König U., Barresi C., Buchberger M., Ghannadan M., Zhang C.-F., Mlitz V., Gmeiner R., Sukseree S., Födinger D. (2013). Epidermal keratinocytes form a functional skin barrier in the absence of Atg7 dependent autophagy. J. Dermatol. Sci..

[42] Koenig U., Robenek H., Barresi C., Brandstetter M., Resch G.P., Gröger M., Pap T., Hartmann C. (2020). Cell death induced autophagy contributes to terminal differentiation of skin and skin appendages. Autophagy.

[43] Rossiter H., Stübiger G., Gröger M., König U., Gruber F., Sukseree S., Mlitz V., Buchberger M., Oskolkova O., Bochkov V. (2018). Inactivation of autophagy leads to changes in sebaceous gland morphology and function. Exp. Dermatol..

[44] Rossiter H., Copic D., Direder M., Gruber F., Zoratto S., Marchetti-Deschmann M., Kremslehner C., Sochorová M., Nagelreiter I.M., Mlitz V. (2022). Autophagy protects murine preputial glands against premature aging, and controls their sebum phospholipid and pheromone profile. Autophagy.

[45] Jaeger K., Sukseree S., Zhong S., Phinney B.S., Mlitz V., Buchberger M., Narzt M.S., Gruber F., Tschachler E., Rice R.H. (2019). Cornification of nail keratinocytes requires autophagy for bulk degradation of intracellular proteins while sparing components of the cytoskeleton. Apoptosis.

[46] Liu W., Li K., Wang G., Yang L., Qu Q., Fan Z., Sun Y., Huang J., Miao Y., Hu Z. (2021). Impairment of autophagy may be associated with follicular miniaturization in androgenetic alopecia by inducing premature catagen. J. Dermatol..

[47] Sukseree S., Bergmann S., Pajdzik K., Sipos W., Gruber F., Tschachler E., Eckhart L. (2018). Suppression of epithelial autophagy compromises the homeostasis of sweat glands during aging. J. Investig. Dermatol..

[48] Sukseree S., Bergmann S., Pajdzik K., Tschachler E., Eckhart L. (2018). Suppression of autophagy perturbs turnover of sequestosome-1/p62 in Merkel cells but not in keratinocytes. J. Dermatol. Sci..

[49] Haruna K., Suga Y., Muramatsu S., Taneda K., Mizuno Y., Ikeda S., Ueno T., Kominami E., Tanida I., Tanida I. (2008). Differentiation-specific expression and localization of an autophagosomal marker protein (LC3) in human epidermal keratinocytes. J. Dermatol. Sci..

[50] Kuma A., Komatsu M., Mizushima N. (2017). Autophagy-monitoring and autophagy-deficient mice. Autophagy.

[51] Kuma A., Hatano M., Matsui M., Yamamoto A., Nakaya H., Yoshimori T., Ohsumi Y., Tokuhisa T., Mizushima N. (2004). The role of autophagy during the early neonatal starvation period. Nature.

[52] Komatsu M., Waguri S., Ueno T., Iwata J., Murata S., Tanida I., Ezaki J., Mizushima N., Ohsumi Y., Uchiyama Y. (2005). Impairment of starvation-induced and constitutive autophagy in Atg7-deficient mice. J. Cell Biol..

[53] Yoshihara N., Ueno T., Takagi A., Oliva Trejo J.A., Haruna K., Suga Y., Komatsu M., Tanaka K., Ikeda S. (2015). The significant role of autophagy in the granular layer in normal skin differentiation and hair growth. Arch. Dermatol. Res..

[54] Tarutani M., Itami S., Okabe M., Ikawa M., Tezuka T., Yoshikawa K., Kinoshita T., Takeda J. (1997). Tissue-specific knockout of the mouse Pig-a gene reveals important roles for GPI-anchored proteins in skin development. Proc. Natl. Acad. Sci. USA.

[55] Turksen K., Kupper T., Degenstein L., Williams I., Fuchs E. (1992). Interleukin 6: Insights to its function in skin by overexpression in transgenic mice. Proc. Natl. Acad. Sci. USA.

[56] Sukseree S., Rossiter H., Mildner M., Pammer J., Buchberger M., Gruber F., Watanapokasin R., Tschachler E., Eckhart L. (2013). Targeted deletion of Atg5 reveals differential roles of autophagy in keratin K5-expressing epithelia. Biochem. Biophys. Res. Commun..

[57] Qiang L., Yang S., Cui Y.H., He Y.Y. (2021). Keratinocyte autophagy enables the activation of keratinocytes and fibroblasts and facilitates wound healing. Autophagy.

[58] Xiao T., Liang J., Li M., Guo Y., Chen S., Ke Y., Gao X., Gu H., Chen X. (2024). ATG5-mediated keratinocyte ferroptosis promotes M1 polarization of macrophages to aggravate UVB-induced skin inflammation. J. Photochem. Photobiol. B Biol..

[59] Noguchi S., Honda S., Saitoh T., Matsumura H., Nishimura E., Akira S., Shimizu S. (2019). Beclin 1 regulates recycling endosome and is required for skin development in mice. Commun. Biol..

[60] Sukseree S., Mildner M., Rossiter H., Pammer J., Zhang C.F., Watanapokasin R., Tschachler E., Eckhart L. (2012). Autophagy in the thymic epithelium is dispensable for the development of self-tolerance in a novel mouse model. PLoS ONE.

[61] Qiang L., Sample A., Shea C.R., Soltani K., Macleod K.F., He Y.Y. (2017). Autophagy gene ATG7 regulates ultraviolet radiation-induced inflammation and skin tumorigenesis. Autophagy.

[62] Ida-Yonemochi H., Otsu K., Irié T., Ohazama A., Harada H., Ohshima H. (2024). Loss of autophagy disrupts stemness of ameloblast-lineage cells in aging. J. Dent. Res..

[63] Huyghe J., Priem D., Van Hove L., Gilbert B., Fritsch J., Uchiyama Y., Hoste E., van Loo G., Bertrand M.J.M. (2022). ATG9A prevents TNF cytotoxicity by an unconventional lysosomal targeting pathway. Science.

[64] Medzhitov R., Schneider D.S., Soares M.P. (2012). Disease tolerance as a defense strategy. Science.

[65] Lim S.H.Y., Hansen M., Kumsta C. (2024). Molecular mechanisms of autophagy decline during aging. Cells.

[66] López-Otín C., Blasco M.A., Partridge L., Serrano M., Kroemer G. (2023). Hallmarks of aging: An expanding universe. Cell.

[67] Yang Y., Kremslehner C., Derdak S., Bauer C., Jelleschitz S., Nagelreiter I.M., Rossiter H., Narzt M.S., Gruber F., Sochorová M. (2022). Consequences of autophagy deletion on the age-related changes in the epidermal lipidome of mice. Int. J. Mol. Sci..

[68] Zhao Y., Zhang C.F., Rossiter H., Eckhart L., König U., Karner S., Mildner M., Bochkov V.N., Tschachler E., Gruber F. (2013). Autophagy is induced by UVA and promotes removal of oxidized phospholipids and protein aggregates in epidermal keratinocytes. J. Investig. Dermatol..

[69] Qiang L., Wu C., Ming M., Viollet B., He Y.Y. (2013). Autophagy controls p38 activation to promote cell survival under genotoxic stress. J. Biol. Chem..

[70] Song X., Narzt M.S., Nagelreiter I.M., Hohensinner P., Terlecki-Zaniewicz L., Tschachler E., Grillari J., Gruber F. (2017). Autophagy deficient keratinocytes display increased DNA damage, senescence and aberrant lipid composition after oxidative stress in vitro and in vivo. Redox Biol..

[71] Megyeri K., Orosz L., Bolla S., Erdei L., Rázga Z., Seprényi G., Urbán E., Szabó K., Kemény L. (2018). Propionibacterium acnes induces autophagy in keratinocytes: Involvement of multiple mechanisms. J. Investig. Dermatol..

[72] Wang Z., Zhou H., Zheng H., Zhou X., Shen G., Teng X., Liu X., Zhang J., Wei X., Hu Z. (2021). Autophagy-based unconventional secretion of HMGB1 by keratinocytes plays a pivotal role in psoriatic skin inflammation. Autophagy.

[73] Pino-Belmar C., Aguilar R., Valenzuela-Nieto G.E., Cavieres V.A., Cerda-Troncoso C., Navarrete V.C., Salazar P., Burgos P.V., Otth C., Bustamante H.A. (2024). An intrinsic host defense against HSV-1 relies on the activation of xenophagy with the active clearance of autophagic receptors. Cells.

[74] Barresi C., Rossiter H., Buchberger M., Pammer J., Sukseree S., Sibilia M., Tschachler E., Eckhart L. (2022). Inactivation of autophagy in keratinocytes reduces tumor growth in mouse models of epithelial skin cancer. Cells.

[75] Ikutama R., Peng G., Tsukamoto S., Umehara Y., Trujillo-Paez J.V., Yue H., Nguyen H.L.T., Takahashi M., Kageyama S., Komatsu M. (2023). Cathelicidin LL-37 activates human keratinocyte autophagy through the P2X_7_, mechanistic target of rapamycin, and MAPK pathways. J. Investig. Dermatol..

[76] Lousada M.B., Edelkamp J., Lachnit T., Fehrholz M., Pastar I., Jimenez F., Erdmann H., Bosch T.C.G., Paus R. (2024). Spatial distribution and functional impact of human scalp hair Follicle microbiota. J. Investig. Dermatol..

[77] Vietri Rudan M., Watt F.M. (2022). Mammalian epidermis: A compendium of lipid functionality. Front. Physiol..

[78] Morioka K., Takano-Ohmuro H., Sameshima M., Ueno T., Kominami E., Sakuraba H., Ihara S. (1999). Extinction of organelles in differentiating epidermis. Acta Histochem. Cytochem..

[79] Simpson C.L., Patel D.M., Green K.J. (2011). Deconstructing the skin: Cytoarchitectural determinants of epidermal morphogenesis. Nat. Rev. Mol. Cell Biol..

[80] Evrard C., Lambert de Rouvroit C., Poumay Y. (2021). Epidermal hyaluronan in barrier alteration-related disease. Cells.

[81] Schmuth M., Eckmann S., Moosbrugger-Martinz V., Ortner-Tobider D., Blunder S., Trafoier T., Gruber R., Elias P.M. (2024). Skin barrier in atopic dermatitis. J. Investig. Dermatol..

[82] Simpson C.L., Tokito M.K., Uppala R., Sarkar M.K., Gudjonsson J.E., Holzbaur E.L.F. (2021). NIX initiates mitochondrial fragmentation via DRP1 to drive epidermal differentiation. Cell Rep..

[83] Petukhova L., Patel A.V., Rigo R.K., Bian L., Verbitsky M., Sanna-Cherchi S., Erjavec S.O., Abdelaziz A.R., Cerise J.E., Jabbari A. (2020). Integrative analysis of rare copy number variants and gene expression data in alopecia areata implicates an aetiological role for autophagy. Exp. Dermatol..

[84] Lin Y., Wu X., Yang Y., Wu Y., Xiang L., Zhang C. (2024). The multifaceted role of autophagy in skin autoimmune disorders: A guardian or culprit?. Front. Immunol..

[85] Klapan K., Frangež Ž., Markov N., Yousefi S., Simon D., Simon H.U. (2021). Evidence for lysosomal dysfunction within the epidermis in psoriasis and atopic dermatitis. J. Investig. Dermatol..

[86] Klapan K., Simon D., Karaulov A., Gomzikova M., Rizvanov A., Yousefi S., Simon H.U. (2022). Autophagy and skin diseases. Front. Pharmacol..

[87] Sukseree S., Karim N., Jaeger K., Zhong S., Rossiter H., Nagelreiter I.M., Gruber F., Tschachler E., Rice R.H., Eckhart L. (2024). Autophagy controls the protein composition of hair shafts. J. Investig. Dermatol..

[88] Kirkin V., Rogov V.V. (2019). A diversity of selective autophagy receptors determines the specificity of the autophagy pathway. Mol. Cell.

[89] Ipponjima S., Umino Y., Nagayama M., Denda M. (2020). Live imaging of alterations in cellular morphology and organelles during cornification using an epidermal equivalent model. Sci. Rep..

[90] Cardoso J.C., Veraitch O., Gianotti R., Ferrara G., Tomasini C.F., Singh M., Zalaudek I., Stefanato C.M. (2017). ‘Hints’ in the horn: Diagnostic clues in the stratum corneum. J. Cutan. Pathol..

[91] Orwin D.F. (1976). Acid phosphatase distribution in the wool follicle. III. Fate of organelles in keratinized cells. J. Ultrastruct. Res..

[92] Fischer H., Szabo S., Scherz J., Jaeger K., Rossiter H., Buchberger M., Ghannadan M., Hermann M., Theussl H.C., Tobin D.J. (2011). Essential role of the keratinocyte-specific endonuclease DNase1L2 in the removal of nuclear DNA from hair and nails. J. Investig. Dermatol..

[93] Liu H., Su P., Li Y., Hoover A., Hu S., King S.A., Zhao J., Guan J.L., Chen S.Y., Zhao Y. (2024). VAMP2 controls murine epidermal differentiation and carcinogenesis by regulation of nucleophagy. Dev. Cell.

[94] Rogerson C., Wotherspoon D.J., Tommasi C., Button R.W., O’Shaughnessy R.F.L. (2021). Akt1-associated actomyosin remodelling is required for nuclear lamina dispersal and nuclear shrinkage in epidermal terminal differentiation. Cell Death Differ..

[95] Moriyama M., Moriyama H., Uda J., Matsuyama A., Osawa M., Hayakawa T. (2014). BNIP3 plays crucial roles in the differentiation and maintenance of epidermal keratinocytes. J. Investig. Dermatol..

[96] Chevalier F.P., Rorteau J., Ferraro S., Martin L.S., Gonzalez-Torres A., Berthier A., El Kholti N., Lamartine J. (2022). MiR-30a-5p alters epidermal terminal differentiation during aging by regulating BNIP3L/NIX-dependent mitophagy. Cells.

[97] Prashar A., Bussi C., Fearns A., Capurro M.I., Gao X., Sesaki H., Gutierrez M.G., Jones N.L. (2024). Lysosomes drive the piecemeal removal of mitochondrial inner membrane. Nature.

[98] Lemasters J.J., Ramshesh V.K., Lovelace G.L., Lim J., Wright G.D., Harland D., Dawson T.L. (2017). Compartmentation of mitochondrial and oxidative metabolism in growing hair follicles: A ring of fire. J. Investig. Dermatol..

[99] Kimura T., Mandell M., Deretic V. (2016). Precision autophagy directed by receptor regulators—Emerging examples within the TRIM family. J. Cell Sci..

[100] Nthiga T.M., Shrestha B.K., Bruun J.A., Larsen K.B., Lamark T., Johansen T. (2021). Regulation of Golgi turnover by CALCOCO1-mediated selective autophagy. J. Cell Biol..

[101] Chino H., Hatta T., Natsume T., Mizushima N. (2019). Intrinsically disordered protein TEX264 mediates ER-phagy. Mol. Cell.

[102] Grumati P., Morozzi G., Hölper S., Mari M., Harwardt M.I., Yan R., Müller S., Reggiori F., Heilemann M., Dikic I. (2017). Full length RTN3 regulates turnover of tubular endoplasmic reticulum via selective autophagy. eLife.

[103] Chen Q., Xiao Y., Chai P., Zheng P., Teng J., Chen J. (2019). ATL3 is a tubular ER-phagy receptor for GABARAP-mediated selective autophagy. Curr. Biol..

[104] Hoyer M.J., Swarup S., Harper J.W. (2022). Mechanisms controlling selective elimination of damaged lysosomes. Curr. Opin. Physiol..

[105] Gahlot P., Kravic B., Rota G., van den Boom J., Levantovsky S., Schulze N., Maspero E., Polo S., Behrends C., Meyer H. (2024). Lysosomal damage sensing and lysophagy initiation by SPG20-ITCH. Mol. Cell.

[106] Waite K.A., Burris A., Vontz G., Lang A., Roelofs J. (2022). Proteaphagy is specifically regulated and requires factors dispensable for general autophagy. J. Biol. Chem..

[107] Date Y., Matsuura A., Itakura E. (2022). Disruption of actin dynamics induces autophagy of the eukaryotic chaperonin TRiC/CCT. Cell Death Discov..

[108] Beese C.J., Brynjólfsdóttir S.H., Frankel L.B. (2020). Selective autophagy of the protein homeostasis machinery: Ribophagy, proteaphagy and ER-phagy. Front. Cell Dev. Biol..

[109] Ciechanover A. (2005). Intracellular protein degradation: From a vague idea, through the lysosome and the ubiquitin-proteasome system, and onto human diseases and drug targeting (Nobel lecture). Angew. Chem. Int. Ed. Engl..

[110] Liu Y., Cui J., Zhang J., Chen Z., Song Z., Bao D., Xiang R., Li D., Yang Y. (2023). Excess KLHL24 impairs skin wound healing through the degradation of vimentin. J. Investig. Dermatol..

[111] Vermeer M.C.S.C., Silljé H.H.W., Pas H.H., Andrei D., van der Meer P., Bolling M.C. (2022). Keratin 14 degradation and aging in epidermolysis bullosa simplex due to KLHL24 gain-of-function variants. J. Investig. Dermatol..

[112] Cui J., Zhao Q., Song Z., Chen Z., Zeng X., Wang C., Lin Z., Wang F., Yang Y. (2022). KLHL24-mediated hair follicle stem cells structural disruption causes alopecia. J. Investig. Dermatol..

[113] Büchau F., Munz C., Has C., Lehmann R., Magin T.M. (2018). KLHL16 degrades epidermal keratins. J. Investig. Dermatol..

[114] Dahlqvist J., Klar J., Tiwari N., Schuster J., Törmä H., Badhai J., Pujol R., van Steensel M.A., Brinkhuizen T., Gijezen L. (2010). A single-nucleotide deletion in the POMP 5′ UTR causes a transcriptional switch and altered epidermal proteasome distribution in KLICK genodermatosis. Am. J. Hum. Genet..

[115] Paul A.A., Szulc N.A., Kobiela A., Brown S.J., Pokrzywa W., Gutowska-Owsiak D. (2023). In silico analysis of the profilaggrin sequence indicates alterations in the stability, degradation route, and intracellular protein fate in filaggrin null mutation carriers. Front. Mol. Biosci..

[116] Briot J., Arbey E., Goudounèche D., Bernard D., Simon M., Méchin M.C. (2024). Human filaggrin monomer does not seem to be a proteasome target. Exp. Dermatol..

[117] Lippens S., Kockx M., Knaapen M., Mortier L., Polakowska R., Verheyen A., Garmyn M., Zwijsen A., Formstecher P., Huylebroeck D. (2000). Epidermal differentiation does not involve the pro-apoptotic executioner caspases, but is associated with caspase-14 induction and processing. Cell Death Differ..

[118] Fischer H., Stichenwirth M., Dockal M., Ghannadan M., Buchberger M., Bach J., Kapetanopoulos A., Declercq W., Tschachler E., Eckhart L. (2004). Stratum corneum-derived caspase-14 is catalytically active. FEBS Lett..

[119] Denecker G., Hoste E., Gilbert B., Hochepied T., Ovaere P., Lippens S., Van den Broecke C., Van Damme P., D’Herde K., Hachem J.P. (2007). Caspase-14 protects against epidermal UVB photodamage and water loss. Nat. Cell Biol..

[120] Zeeuwen P.L. (2004). Epidermal differentiation: The role of proteases and their inhibitors. Eur. J. Cell Biol..

[121] Peled A., Sprecher E. (2024). Proteolytic and antiproteolytic activity in the skin: Gluing the pieces together. J. Investig. Dermatol..

[122] Fischer H., Buchberger M., Napirei M., Tschachler E., Eckhart L. (2017). Inactivation of DNase1L2 and DNase2 in keratinocytes suppresses DNA degradation during epidermal cornification and results in constitutive parakeratosis. Sci. Rep..

[123] Manils J., Fischer H., Climent J., Casas E., García-Martínez C., Bas J., Sukseree S., Vavouri T., Ciruela F., de Anta J.M. (2017). Double deficiency of Trex2 and DNase1L2 nucleases leads to accumulation of DNA in lingual cornifying keratinocytes without activating inflammatory responses. Sci. Rep..

[124] Monteleon C.L., Agnihotri T., Dahal A., Liu M., Rebecca V.W., Beatty G.L., Amaravadi R.K., Ridky T.W. (2018). Lysosomes support the degradation, signaling, and mitochondrial metabolism necessary for human epidermal differentiation. J. Investig. Dermatol..

[125] Hailfinger S., Schulze-Osthoff K. (2021). Impaired autophagy in psoriasis and atopic dermatitis: A new therapeutic target?. J. Investig. Dermatol..

[126] Neto M.V., Hall M.J., Charneca J., Escrevente C., Seabra M.C., Barral D.C. (2024). Photoprotective melanin is maintained within keratinocytes in storage lysosomes. J. Investig. Dermatol..

[127] Wang F., Gómez-Sintes R., Boya P. (2018). Lysosomal membrane permeabilization and cell death. Traffic.

[128] Meyer H., Kravic B. (2024). The endo-lysosomal damage response. Annu. Rev. Biochem..

[129] Fischer H., Scherz J., Szabo S., Mildner M., Benarafa C., Torriglia A., Tschachler E., Eckhart L. (2011). DNase 2 is the main DNA-degrading enzyme of the stratum corneum. PLoS ONE.

[130] Kroemer G., Jäättelä M. (2005). Lysosomes and autophagy in cell death control. Nat. Rev. Cancer.

[131] Fukuda K., Ito Y., Furuichi Y., Matsui T., Horikawa H., Miyano T., Okada T., van Logtestijn M., Tanaka R.J., Miyawaki A. (2024). Three stepwise pH progressions in stratum corneum for homeostatic maintenance of the skin. Nat. Commun..

[132] Murata T., Honda T., Egawa G., Yamamoto Y., Ichijo R., Toyoshima F., Dainichi T., Kabashima K. (2018). Transient elevation of cytoplasmic calcium ion concentration at a single cell level precedes morphological changes of epidermal keratinocytes during cornification. Sci. Rep..

[133] Murase D., Kusaka-Kikushima A., Hachiya A., Fullenkamp R., Stepp A., Imai A., Ueno M., Kawabata K., Takahashi Y., Hase T. (2020). Autophagy declines with premature skin aging resulting in dynamic alterations in skin pigmentation and epidermal differentiation. Int. J. Mol. Sci..

[134] Seo S.H., Jung J.Y., Park K., Hossini A.M., Zouboulis C.C., Lee S.E. (2020). Autophagy regulates lipid production and contributes to the sebosuppressive effect of retinoic acid in human SZ95 sebocytes. J. Dermatol. Sci..

[135] Wang D., Jiang J., Wang M., Li K., Liang H., Wang N., Liu W., Wang M., Zhou S., Zhang M. (2024). Mitophagy promotes hair regeneration by activating glutathione metabolism. Research.

[136] Gund R., Christiano A.M. (2023). Impaired autophagy promotes hair loss in the C3H/HeJ mouse model of alopecia areata. Autophagy.

[137] Jin X., Song X. (2024). Autophagy dysfunction: The kernel of hair loss?. Clin. Cosmet. Investig. Dermatol..

[138] Chai M., Jiang M., Vergnes L., Fu X., de Barros S.C., Doan N.B., Huang W., Chu J., Jiao J., Herschman H. (2019). Stimulation of hair growth by small molecules that activate autophagy. Cell Rep..

